# Diminished neutralizing activity against the XBB1.5 strain in 55.9% of individuals post 6 months COVID-19 mRNA booster vaccination: insights from a pseudovirus assay on 1,353 participants in the Fukushima vaccination community survey, Japan

**DOI:** 10.3389/fimmu.2024.1337520

**Published:** 2024-03-18

**Authors:** Tianchen Zhao, Yuta Tani, Chieko Makino-Okamura, Morihito Takita, Chika Yamamoto, Eiki Kawahara, Toshiki Abe, Sota Sugiura, Hiroki Yoshimura, Taiga Uchiyama, Isato Yamazaki, Harumichi Ishigame, Takaharu Ueno, Kazu Okuma, Masatoshi Wakui, Hidehiro Fukuyama, Masaharu Tsubokura

**Affiliations:** ^1^ General Incorporated Association for Comprehensive Disaster Health Management Research Institute, Tokyo, Japan; ^2^ Department of Radiation Health Management, Fukushima Medical University School of Medicine, Fukushima, Fukushima, Japan; ^3^ Department of Laboratory Medicine, Keio University School of Medicine, Tokyo, Japan; ^4^ Division of Immunology, Near-InfraRed Photo-Immunotherapy Research Institute, Kansai Medical University, Hirakata, Osaka, Japan; ^5^ Infectious Diseases Research Unit, RIKEN Center for Integrative Medical Sciences, Yokohama, Kanagawa, Japan; ^6^ Cell Integrative Science Laboratory, Graduate School of Medical Life Science, Yokohama City University, Yokohama, Kanagawa, Japan; ^7^ Laboratory for Tissue Dynamics, RIKEN Center for Integrative Medical Sciences, Yokohama, Kanagawa, Japan; ^8^ Department of Microbiology, Kansai Medical University, School of Medicine, Hirakata, Osaka, Japan; ^9^ INSERM EST, Strasbourg, France

**Keywords:** SARS-CoV2, vaccination, antibody waning, bivalent vaccination, mRNA vaccination vulnerable population

## Abstract

This study investigates the neutralizing activity against the XBB1.5 variant and the ancestral strain in a population post-bivalent vaccination using a pseudo virus assay validated with authentic virus assay. While bivalent booster vaccination and past infections enhanced neutralization against the XBB 1.5 strain, individuals with comorbidities showed reduced responses. The study suggests the need for continuous vaccine updates to address emerging SARS-CoV-2 variants and highlights the importance of monitoring real-world immune responses.

## 1 Introduction

The enhancement of immunity, resulting from both vaccination and natural infection (known as “hybrid immunity”), has remarkedly decreased mortality rates from the severe acute respiratory syndrome coronavirus 2 (SARS-CoV-2), transitioning the situation from a pandemic to an endemic. Nevertheless, the importance of continuously updating vaccinations to address the latest viral variants cannot be overstated. Studies have indicated a significant decline in hybrid immunity over time in those aged 65 and over, and new Variants of Concern (VOCs) such as EG.5, FL.1.5.1, and BA.2.86, have emerged in fall 2023 (1).

As regularly updating vaccines to match prevalent variants becomes a standard approach, it is crucial to monitor immune responses, including neutralizing capabilities, in the real world. Numerous studies employing pseudo virus assays have reported a significant evasion of neutralization capability against the Omicron subvariant, particularly XBB1.5, even after administering a bivalent vaccine booster (2–6). However, few studies exist on the waning of neutralization against XBB1.5 following month after bivalent vaccination and on demographics that exhibit diminished neutralization capabilities. Thus, we conducted a neutralization assay using pseudo viruses to evaluate the response against both the Wuhan strain and the XBB1.5 strain at the population level in participants from the Fukushima vaccination cohort (7), incorporating detailed profiling data of medical and medication histories.

## 2 Material and methods

### 2.1 For participant eligibility and sample collection

Study participants were recruited from Hirata village, Soma City, and Minamisoma city, located in the rural region of Fukushima Prefecture, Japan. They received their first and second doses of the BNT162b2 vaccine (Pfizer/BioNTech, New York, USA). For those receiving subsequent doses, monovalent vaccine recipients were administered either BNT162b2 (Pfizer-BioNTech) or mRNA1273 (Moderna, Cambridge, MA, USA). For individuals receiving bivalent vaccinations, their options included Comirnaty Bivalent Original/Omicron BA.1/BA.2 (Pfizer/BioNTech, New York, USA), Comirnaty Bivalent Original/Omicron BA.4/BA.5 (Pfizer/BioNTech), Spikevax Bivalent Original/Omicron BA.1/BA.2 (Moderna, Cambridge, MA, USA), or Spikevax Bivalent Original/Omicron BA.4/BA.5 (Moderna). Bivalent vaccination in Japan commenced on September 20, 2022. This study included survey participants who completed at least two initial doses of the mRNA BNT162b2 vaccine. Blood collections for this study were performed in March and April, 2023, detailed is referred to **Supplementary Figure 1**. Collected whole blood and serum samples were forwarded to the Kansai University (Osaka, Japan) and the University of Tokyo (Tokyo, Japan) for SARS-CoV-2 pseudo virus neutralization assay and the assessment of SARS-CoV-2 specific nucleocapsid antibodies, respectively. Demographic and health-related data, such as age, sex, the interval between vaccination and blood collection, vaccine type, smoking and drinking habits, and comorbidities, were gleaned from paper-based questionnaires.

### 2.2 Measurement of SARS-CoV-2-specific spike protein and nucleocapsid antibody

The details are descried in our past paper (8). Briefly, SARS-CoV-2 specific spike protein IgG (IgG(S)) and nucleocapsid antibody IgG (IgG(N)) were measured. If the titers of IgG(N) is higher than 10.0 AU/mL, we regard it as a past infection. Chemiluminescent immunoassays using iFlash 3000 (YHLO Biotech, Shenzhen, China) and iFlash-2019-nCoV series (YHLO Biotech) reagents were used in the present study. All testing processes followed the official guidelines. Quality checks were conducted every day before measurements.

### 2.3 SARS-CoV-2 pseudovirus neutralization assay

Pseudovirus neutralization assay was performed as previously described (9). Briefly, two plasmids, pNL4-3.luc.R-E- and pCMV3_SARS-cov2d19 series generated from Spike expression plasmid(hCoV-19/Wuhan/WIV04/2019 (WIV04): EPI_ISL_402124 as Wuhan and Pango Lineage: XBB.1.5 (Pango v.4.3.1 consensus call), Omicron (XBB.1.5-like) (Scorpio): EPI_ISL_16826532 as XBB1.5), were transfected into cells and the pseudovirus was produced using the Expi293 expression system (Thermo Fisher Scientific); those viruses were used for the assay. After series of diluted serum/plasma samples using iDOT (Cytena) were mixed with 3000 units/well viruses of each strain in 10 μL/well with D-MEM containing 10% FCS, 1× MEM non-essential amino acids solution, and 1 mM sodium pyrubate, incubated for 37°C for 30 min, 6000 cells/10μL/well of human ACE2; TMPRSS2-expressing 293FT cell line (DSP1-736) were added in a 384-well plate (BioTec, EDR-24LX and -384SR). After 48 h, 20 μL/well of ONE-Glo™ EX Reagent was added (ONE-Glo™ EX Luciferase Assay Sytem, Promega). After 5 min agitation, luminescence was measured using a GloMax^®^ Discover microplate reader (Promega). The data from biological duplicate were analyzed using GraphPad Prism (GraphPad software ver, 10.0.2) for determining the value of ID_50_ and ID_80_. Typical inhibition curves of neutralization assays and heatmap of ID50 and ID80 are shown in **Supplementary Figure 2**.

### 2.4 SARS-CoV-2 authentic virus neutralization assay

Authentic virus neutralization assay was performed as previously described (9). Briefly, 14,000 cells/well VeroE6/TMPRSS2 were seeded in a 96-well plate 1 day before infection. Serially diluted sera and 100 TCID50 viruses of for Wuhan strain or the XBB1.15.19 variants, were mixed in D-MEM Low glucose containing 2% FCS incubated for 37°C for 1h and applied to the cell layers. After 5 days, CPEs were observed under an inverted microscope and the value of ID50 were determined by the Reed-Muench method. The authentic virus utilized in this study was supplied by the National Institute of Infectious Diseases. The 19 samples were selected targeted the quartile range (Q1, Q2, Q3) of titers against the Wuhan strain in the pseudo virus neutralization assay. From those without a history of infection, 6 specimens were picked from Q1, 7 specimens from Q2, and 6 specimens from Q3.

### 2.5 Statistics analysis

A descriptive analysis was conducted. The continuous and categorical variables were summarized as median (interquartile range) and numbers (percentages), respectively. An univariable and multivariable ordinal regression analysis was performed to determine factors associated with higher neutralizing activity against the Wuhan and XBB1.5 strains. The neutralizing activity was quantified using natural log values, and subsequently categorized into nine distinct tiers for ordinal ranking. We used patient characteristics (age, smoking habit, drinking habit, comorbidity, days from the last vaccination, type of the last vaccination, and past infection as the explanatory variables. We conducted Spearman’s correlation analysis to investigate the presence of confounding among the variables. The IBM SPSS Statistics (IBM ver. 28.0.1.0) software and GraphPad Prism (GraphPad software version 10.0.2) were used for all analysis and figures.

## 3 Results

### 3.1 Characteristics of participants

This study included a total of 1,353 participants (**Table 1**). The median age was 50 years, and 833 (61.6%) individuals were female. 719 (53.1%) had comorbidities, including hypertension and diabetes mellitus. The past infection defined by IgG(N) ≥10.0 AU/mL was found in 253 participants (18.7%). 1,172 (86.6%) received a bivalent booster. The median duration between the last vaccination and blood collection was 143 days (interquartile range: 118–168). The median titer of serum IgG(S) was 2137.6 AU/mL (interquartile range: 1103.2–4028.4). The detailed information about the timeline of blood collection and the vaccination are shown in **Supplementary Figure 1**.

**Table 1:** Results of ordered logistic regression analysis.

Variables	N = 1353	Wuhan				XBB 1.5			
		Univariable analysis		Multivariable analysis		Univariable analysis		Multivariable analysis	
		OR (95% CI)	p-value	aOR (95% CI)	p value	OR (95% CI)	p-value	aOR (95% CI)	p value
Age	50 [39-63]								
- 40 years old	376(27.8)	1(ref)	–	1(ref)	–	1(ref)	–	1(ref)	–
41 - 60 years old	585(43.2)	1.10 (0.87—1.38)	0.42	1.01 (0.77—1.32)	0.95	0.81 (0.65—1.02)	0.07	0.86 (0.66—1.12)	0.25
61- years old	392(29.0)	1.01 (0.79—1.30)	0.94	1.05 (0.77—1.44)	0.75	0.61 (0.47—0.78)	<0.001	0.70 (0.27—1.76)	0.44
Smoking	210(15.5)	0.88 (0.68—1.15)	0.35	0.90 (0.68—1.18)	0.44	1.01 (0.78—1.32)	0.93	0.93 (0.71—1.22)	0.60
Drinking Habit	480(35.5)	0.84 (0.68—1.04)	0.10	0.86 (0.7—1.07)	0.18	0.85 (0.69—1.04)	0.12	0.89 (0.72—1.11)	0.31
Comorbidity	719(53.1)	0.92 (0.76—1.11)	0.37	1.00 (0.8—1.25)	0.99	0.74 (0.61—0.89)	<0.001	0.76 (0.61—0.96)	0.02
Days from the last vaccination	143 [118-168]								
- 3 months	82(6.1)	1(ref)	–	1(ref)	–	1(ref)	–	1(ref)	–
3 - 6 months	1079(79.7)	0.37 (0.26—0.54)	<0.001	0.43 (0.28—0.67)	<0.001	0.53 (0.36—0.77)	<0.001	0.66 (0.43—1.01)	0.06
6- months	183(13.5)	0.40 (0.26—0.63)	<0.001	0.38 (0.15—0.97)	0.04	0.59 (0.38—0.92)	0.02	0.70 (0.27—1.76)	0.44
Type of the last vaccination	*1missing value								
Pfizer monovalent	98(7.2)	1(ref)	–	1(ref)	–	1(ref)	–	1(ref)	–
Pfizer BA.1/BA.2	98(7.2)	2.30 (1.40—3.76)	<0.001	1.52 (0.60—3.90)	0.38	2.88 (1.76—4.71)	<0.001	3.41 (1.33—8.73)	0.01
Pfizer BA.4/BA.5	933(69.0)	1.03 (0.71—1.49)	0.86	0.85 (0.34—2.12)	0.73	1.12 (0.78—1.62)	0.54	1.20 (0.48—2.99)	0.70
Moderna monovalent	82(6.1)	1.33 (0.79—2.22)	0.28	1.06 (0.59—1.93)	0.84	1.80 (1.08—3.01)	0.03	1.75 (0.97—3.16)	0.06
Moderna BA.1/BA.2	127(9.4)	2.40 (1.50—3.83)	<0.001	2.05 (0.79—5.38)	0.14	3.89 (2.44—6.20)	<0.001	4.31 (1.65—11.31)	<0.001
ModernaBA.4/BA.5	14(1.0)	0.87 (0.33—2.33)	0.79	0.78 (0.21—2.96)	0.72	1.73 (0.65—4.60)	0.27	1.99 (0.52—7.51)	0.31
Past Infection IgG(N) ≧10.0 AU/mL	253(18.7)	3.15 (2.46—4.02)	<0.001	3.36 (2.53—4.46)	<0.001	2.00 (1.57—2.54)	<0.001	1.77 (1.34—2.33)	<0.001

Median [interquartile] or number (percentage) are shown for continuous or categorical variables. 95% CI, 95% confidence interval; aOR, adjusted odds ratio; OR, odds ratio.

### 3.2 Validation of pseudo virus neutralization assay using SARS-CoV-2 authentic virus neutralization assay

The results of plotting the SARS-CoV-2 pseudo virus and authentic virus neutralization titers are presented in **Supplementary Figures 3** and **4**. For the Wuhan strain, a significantly high correlation was demonstrated with square of a correlation coefficient (*R^2^*) of 0.801 and a *p* value < 0.0001 after excluding an outlier of samples defined by the robust regression and outlier removal (ROUT) method. For the XBB1.5 strain, an approximately significant high correlation was shown with *R^2^* of 0.623 and a *p* value of 0.0044. The results of plotting IgG(S) with pseudo virus and authentic virus neutralization activity are presented in **Supplementary Figure 5**. For the pseudo virus, a significant high correlation was demonstrated with an *R^2^* of 0.7665 and a *p* value < 0.0001. For the authentic virus, a significantly high correlation was shown with an *R^2^* of 0.6044 and a *p* value of 0.0001.

### 3.3 Neutralizing responses against the Wuhan strain and XBB1.5 strain

**Figure 1:**
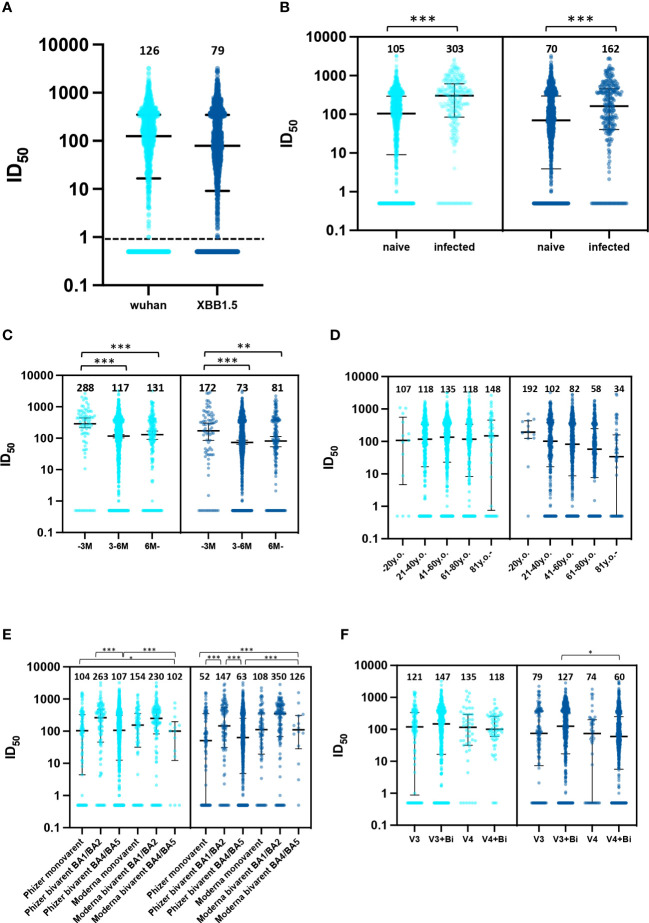
Neutralizing responses against the Wuhan strain and XBB1.5 strain. **(A)** distribution of neutralizing responses in the entire cohort, **(B)** comparative distribution of neutralizing responses: naive vs. previously infected, **(C)** distribution of neutralizing responses relative to time elapsed since last vaccination, months; M, **(D)** age-stratified distribution of neutralizing responses, years old; y.o., **(E)** vaccine type-specific distribution of neutralizing responses and **(F)** distribution of neutralizing responses by number of vaccine doses administered. 126 participants (9.3%) received three doses of an ancestral-strain monovalent vaccine (V3 group). 467 (37.5%) received three doses of a monovalent vaccine and one dose of a bivalent vaccine (V3+Bi group), 56 (4.1%) were administered four doses of the monovalent vaccine (V4 group), and 704 (52.0%) received four doses of a monovalent vaccine and one dose of a bivalent vaccine (V4+Bi group). *; p<0.05, **; p<0.01, ***; p<0.001.

The medians of neutralizing titer against the Wuhan and XBB strains in our cohort were 125.6 and 79.0, respectively (**Figure 1A**). 44.6% (593/1,331) and 54.8% (731/1,334) demonstrated an ID50 value at or below 100 with pseudo viruses of ancestral and XBB strains, respectively. Individuals with past infection showed higher median titers in comparison to those uninfected (**Figure 1B**). When stratified by duration after the last vaccination, the neutralizing titer against both the ancestral and XBB1.5 strains revealed decreased responses in the 3–6 months period in comparison with the first 3 months post-vaccination. Neutralization titers were shown after classifying the groups of age and vaccination types (**Figures 1C, F**). Age and post-vaccination durations were unevenly distributed based on the vaccination types within this cohort (**Supplementary Figure 6**).

### 3.4 Ordinal regression analysis: patient characteristics correlating with elevated and diminished neutralization against the Wuhan and XBB1.5 strains

Multivariate analysis revealed that a post-vaccination duration of 3–6 months and six months or longer, compared to within 3 months, had an inverse effect on the ancestral strain neutralization (adjusted odds ratio; aOR [95% CI]: 0.43 [0.28–0.67], p <0.001, and 0.38 [0.15–0.97], p =0.04, respectively) (**Table 1**). A past infection was associated with an increase in neutralization activity (aOR: 3.36 [2.53-4.36], p <0.001). Neutralization of XBB 1.5 strain was enhanced by Pfizer BA.1.BA.2, Moderna BA.1/BA.2, and past infection (aOR [95% CI], p value: 3.41 [1.33–8.73], 0.01; 4.31 [1.65–11.31], <0.001; and 1.77 [1.34–2.33], <0.001, respectively). Comorbidity was negatively correlated with XBB 1.5 strain neutralization (0.76 [0.61–0.96], 0.02). No significant impact of post-vaccination duration on the XBB 1.5 strain neutralization was observed. The results of Spearman’s correlation analysis for variables in ordered logistic regression are presented in **Supplementary Table 1**. Notably, a significant negative correlation was observed between age and days from the last vaccination, with a correlation coefficient of -0.176.

### 3.5 Characteristics of individuals with a history of infection yet exhibiting minimal neutralizing titers against the Wuhan and XBB1.5 strains

Next, we depicted the distribution of neutralizing activity against the Wuhan and XBB strains through heatmaps and calculated average values for age, days after the last vaccination, and IgG(N) levels (**Supplementary Figures 7–10**). 51 participants (20.2%) with a history of infection demonstrated minimal neutralizing titers (ID50 of 100 or less) against either the Wuhan or XBB1.5 strains. 41 (80.4%) of them had received a bivalent vaccination. Seven participants (13.7%) exhibited almost negligible neutralizing titers against both the ancestral and XBB1.5 strains (**Supplementary Table 2**). Of these, all were under 50 years of age and had no comorbidities, with the exception of one individual with 80 years.

## 4 Discussion

To the best of our knowledge, this study is the first to investigate the neutralization capacity using a pseudo virus assay against XBB1.5 at a population level post-bivalent vaccine booster administration. Our findings on the waning neutralization against the XBB1.5 strain align with previously reported data (10, 11). In comparisons, between monovalent and bivalent vaccines, existing literature suggests that bivalent formulations exhibit enhanced efficacy against various VOCs, a finding consistent with our present results (3).

Our results from multivariate regression models are consistent with previous studies except for age. While reduced neutralization is linked to atypical B cells in older populations (12), our study did not find age to be a significant variable. Although caution is essential, the depiction in **Figure 1** of a discernible decrease in neutralization efficacy against the XBB1.5 strain among individuals over 61 years old may be attributed to the notable negative association between age and days since the last vaccination, as evidenced in **Supplementary Table 1**. This might be because the national policy that prioritized vaccination for older individuals, causing potential confounding factors related to post-vaccination duration and vaccine type. Previous studies support our results that past infections were associated with enhanced neutralization activity (13, 14). Our data, indicating superior neutralization by BA.1/BA.2 bivalent booster vaccination over Pfizer monovalent, aligns with the findings of Carr et al. They observed bivalent mRNA BA.1 vaccination exhibiting robust cross-neutralization against emerging omicron subvariants, including XBB.1.5 (15). Of note, even individuals with a history of bivalent vaccination or previous infection may show little to no neutralizing activity. In-depth immunological assessment is needed for these individuals.

This study presents several limitations. Firstly, due to policy-driven vaccination strategies, there were biases in age and post-vaccination period across groups, which limits the comparability, as shown in **Supplementary Table 1**. Secondly, it should be noted that measurements for Variants of Concern (VOCs) other than XBB1.5, such as the parent BA.2 sub lineages, have not been conducted. Thirdly, measurements of the pseudo virus before and after the booster have not been conducted. Fourthly, we could not evaluate the severity of COVID-19 symptoms, such as hospitalization, oxygen supplementation, and death, as endpoints. Therefore, we could not assess the relationship between neutralizing activity and disease severity. Furthermore, we were unable to identify specific strains of past infection or infections caused by seasonal coronaviruses. Lastly, the cohort in the Fukushima Vaccination Community Survey exhibited a low incidence of COVID-19 infection, with 253 out of 1,353 (18.7%) being infected during March and April 2023. This poses challenges in evaluating the hard endpoints of hospitalization and death; however, it may benefit in assessing the effect of vaccinations in uninfected individuals. The Fukushima Vaccine Cohort can provide insights on vaccine-derived immunity in a large demographic over two years.

## Data availability statement

The original contributions presented in the study are included in the article/**Supplementary Materials**. Further inquiries can be directed to the corresponding authors.

## Ethics statement

The studies involving humans were approved by Hirata Central Hospital (number 2021-0611-1) and Fukushima Medical University School of Medicine (number 2021-116). The studies were conducted in accordance with the local legislation and institutional requirements. The participants provided their written informed consent to participate in this study. Written informed consent was obtained from the individual(s) for the publication of any potentially identifiable images or data included in this article.

## Author contributions

TZ: Conceptualization, Data curation, Methodology, Project administration, Writing – review & editing. YT: Data curation, Formal analysis, Investigation, Software, Visualization, Writing – original draft. CM: Conceptualization, Data curation, Methodology, Writing – review & editing. MTa: Conceptualization, Data curation, Formal analysis, Software, Validation, Writing – review & editing. CY: Data curation, Methodology, Project administration, Validation, Writing – review & editing. EK: Methodology, Project administration, Writing – review & editing. TA: Data curation, Project administration, Writing – review & editing. SS: Data curation, Investigation, Project administration, Writing – review & editing. HY: Data curation, Project administration, Writing – review & editing. TUc: Project administration, Writing – review & editing. IY: Project administration, Writing – review & editing. HI: Project administration, Writing – review & editing. TUe: Methodology, Writing – review & editing. KO: Methodology, Writing – review & editing. MW: Supervision, Writing – review & editing. HF: Conceptualization, Methodology, Project administration, Supervision, Writing – original draft. MTs: Funding acquisition, Project administration, Writing – review & editing.
