# Supplementary material for: Diminished neutralizing activity against the XBB1.5 strain in 55.9% of individuals post 6 months COVID-19 mRNA booster vaccination: insights from a pseudovirus assay on 1,353 participants in the Fukushima vaccination community survey, Japan
Source: Front Immunol. 2024 Mar 18;15:1337520. doi: 10.3389/fimmu.2024.1337520 (PMC10983612; doi:10.3389/fimmu.2024.1337520)

Diminished neutralizing activity against the XBB1.5 strain in 55.9% of individuals post 6 months COVID-19 mRNA booster vaccination: Insights from a pseudovirus assay on 1,353 participants in the Fukushima Vaccination Community Survey, Japan

Tianchen Zhao1,2*& Yuta Tani1,3* & Chieko Makino-Okamura4,5*, Morihito Takita1,2, Chika Yamamoto1,2, Eiki Kawahara4,5,6, Toshiki Abe2, Sota Sugiura1, Hiroki Yoshimura1,2, Taiga Uchiyama2, Isato Yamazaki4,5,6, Harumichi Ishigame 4,8, Takaharu Ueno 9, Kazu Okuma 9, Masatoshi Wakui3, Hidehiro Fukuyama4,5,6,7*& Masaharu Tsubokura1,2*

[1] General Incorporated Association for Comprehensive Disaster Health Management Research Institute, Tokyo, Japan

[2] Department of Radiation Health Management, Fukushima Medical University School of Medicine, Fukushima, Japan

[3] Department of Laboratory Medicine, Keio University School of Medicine, Tokyo, Japan

[4] Division of Immunology, Near-InfraRed Photo-Immunotherapy Research Institute, Kansai Medical University, Hirakata, Osaka, 573-1010, Japan

[5] Infectious Diseases Research Unit, RIKEN Center for Integrative Medical Sciences, Kanagawa, Japan.

[6] Cell Integrative Science Laboratory, Graduate School of Medical Life Science, Yokohama City University, Kanagawa, Japan.

[7] INSERM EST, 67037 Strasbourg Cedex 2, France.

[8] Laboratory for Tissue Dynamics, RIKEN Center for Integrative Medical Sciences, Kanagawa, Japan.

[9] Department of Microbiology, Kansai Medical University, School of Medicine, Hirakata, Osaka, 573-1010, Japan.

[2]Laboratory X, Institute X, Department X, Organization X, City X, State XX (only USA, Canada and Australia), Country

*** Correspondence:**
Corresponding Author Hidehiro Fukuyama and Masaharu Tsubokura
email@uni.edu Hidehiro.fukuyama@gmail.com and tsubo-m@fmu.ac.

1. Supplementary Tables and Figures

Supplementary Table 1. Results of Spearman's correlation analysis for variables in ordered logistic regression

Variables	Wuhan	XBB1.5	Age	Smoking	Drinking	Comorbidity	Days from the last vaccination	Type of the last vaccination	Past infection
Wuhan	-	-	-	-	-	-	-	-	-
XBB1.5	**.400****	-	-	-	-	-	-	-	-
Age	0.001	**.096****	-	-	-	-	-	-	-
Smoking	0.033	0.004	-0.036	-	-	-	-	-	-
Drinking	0.049	0.049	0.003	**.096****	-	-	-	-	-
Comorbidity	0.032	**.087****	**.255****	-0.048	0.002	-	-	-	-
Days from the last vaccination	**.069****	0.036	**-.176****	0.012	-0.001	-0.03	-	-	-
Type of the last vaccination	0.015	0.043	**.208****	-0.007	-0.006	0.051	**-.540****	-	-
Past Infection	**-.226****	**-.153****	**-.122****	**-.061***	-0.015	**-.052***	**.056***	**-.099****	-

*; p<0.05, **; p <0.01, ***; p <0.001

Supplementary Table 2. Characteristics of individuals with a bivalent vaccination and a history of infection yet exhibiting minimal neutralizing titers against the Wuhan and XBB1.5 strains

ID	sex	age	BMI	medication	comorbidtiy	group	The type of the last vaccination	Blood collection date	Days elapsed from the last vaccination	IgG(N) [AU/mL]	Neutralizing titers	
											Wuhan strain [IC50]	XBB1.5 strain [IC50]
2790	female	24	missing	none	missing	V3+Bi	Moderna BA.1/BA.2	2023/4/4	171	34.31	0	0
2782	female	30	19.0	none	none	V3+Bi	Pfizer BA.4/BA.5	2023/4/5	132	29.42	0	0
2831	male	31	23.6	none	none	V3	Pfizer monovalent	2023/3/29	429	25.95	0	0
665	female	33	18.4	NSAIDs	none	V3	Moderna monovalent	2023/4/18	435	24.43	0	0
691	female	34	26.2	none	none	V3+Bi	Pfizer BA.4/BA.5	2023/4/17	146	26.21	0	0
685	female	48	20.4	none	none	V3	Pfizer monovalent	2023/4/17	426	134.59	0	0
190	female	80	24.7	none	HT, DLP, OSA, DVT	V4+Bi	Pfizer BA.4/BA.5	2023/3/27	110	15.62	0	0

HT, hypertention; DLP, Dyslipidemia; OSA, Sleep Apnea Syndrome or Obstructive Sleep Apnea; DVT, Deep Vein Thrombosis

Supplementary Figure 1. Timeline of the blood collection and the vaccination

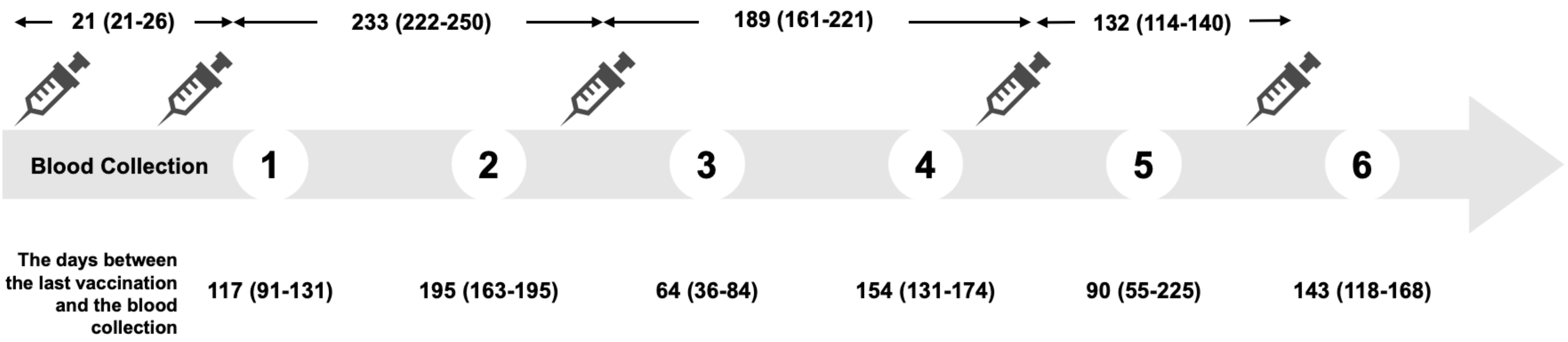

The syringe icon in the figure indicates the timing of vaccination, and the numbers represent the frequency of blood collection. The results presented in this paper correspond to the outcome of the sixth blood collection. The numbers in the figure represent the median (Q1-Q3). It is important to note that not everyone has received the vaccine at these specific times; this is a schematic representation.

Supplementary Figure 2. Typical inhibition curves of neutralization assays and heatmap of ID50 and ID80

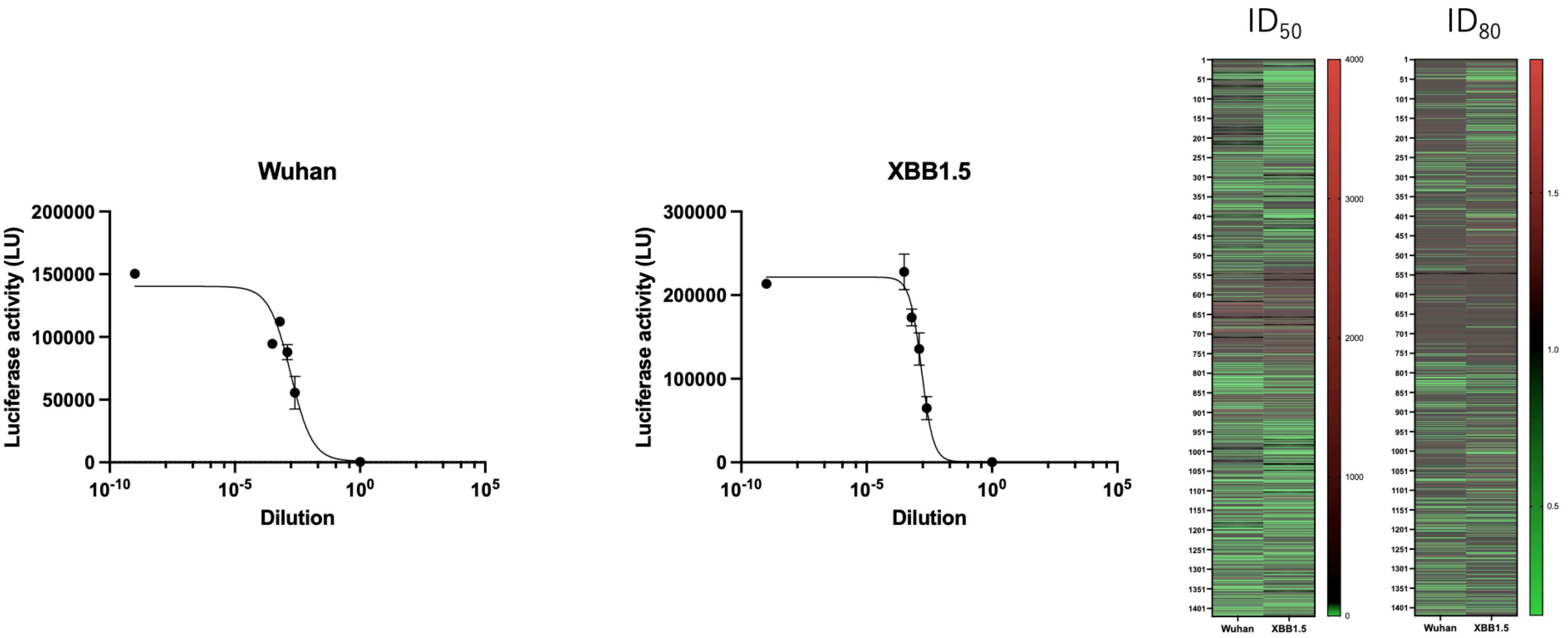

Supplementary Figure 3. Correlation between Wuhan authentic virus and pseudo virus neutralization activity (ID50)

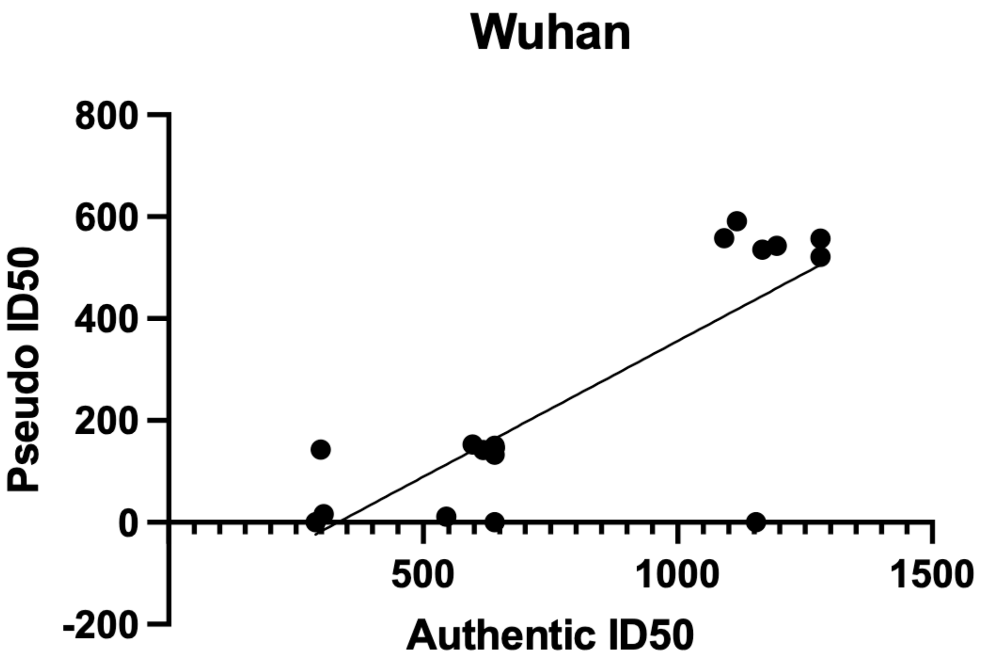

Pearson r	
r	0.8012
95% confidence interval	0.5341 to 0.9229
R squared	0.6419
P value	
P (two-tailed)	<0.0001
P value summary	****
Significant? (alpha = 0.05)	Yes
Number of XY Pairs	18

Eighteen human samples were plotted. One outlier sample was excluded by ROUT.

Supplementary Figure 4. Correlation between XBB1.5 authentic virus and pseudo virus neutralization activity (ID_{50})

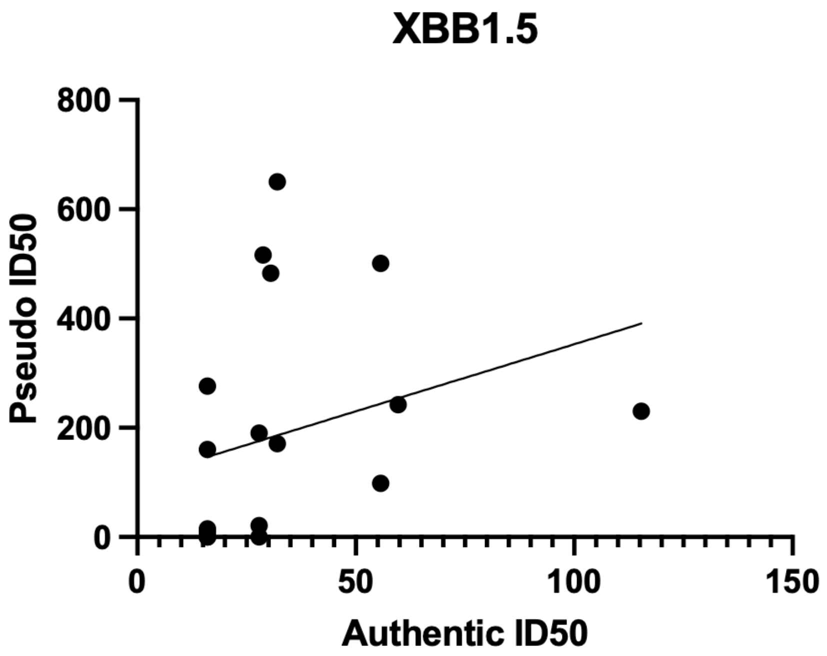

Spearman r	
r	0.6229
95% confidence interval	0.2215 to 0.8438
P value	
P (two-tailed)	0.0044
P value summary	**
Exact or approximate P value?	Approximate
Significant? (alpha = 0.05)	Yes
Number of XY Pairs	19

Nineteen human samples were plotted.

Supplementary Figure 5. Correlation between IgG(S) and Pseudo virus neutralization activity, Wuhan authentic virus neutralization activity

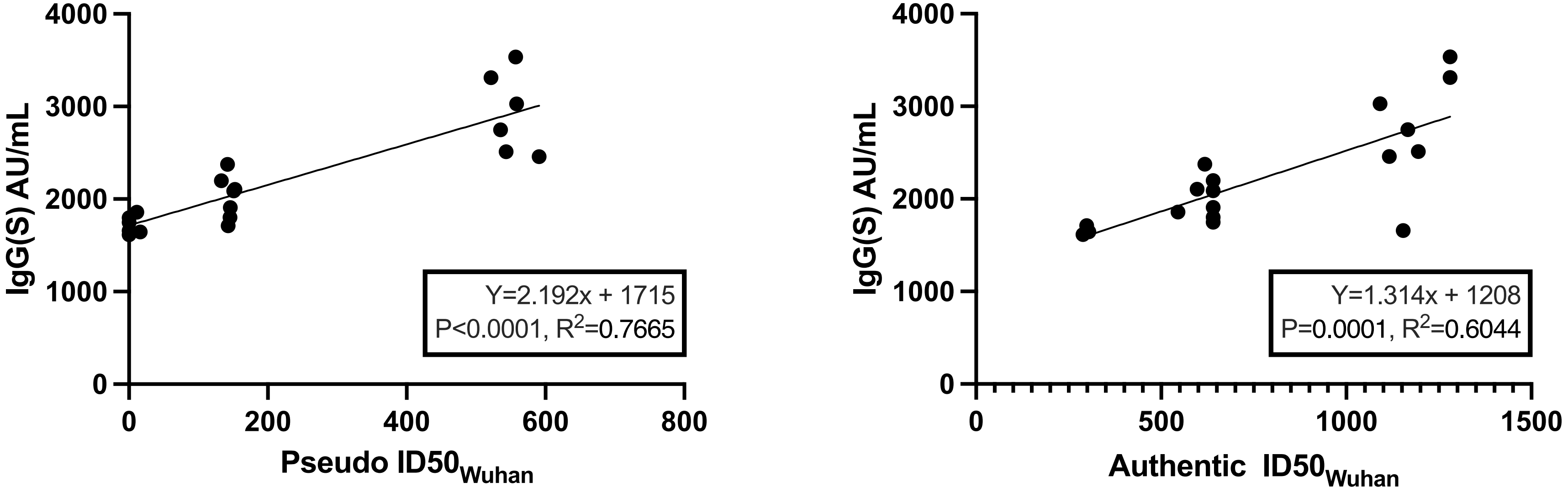

The scatter plot on the left for pseudo-virus neutralization activity includes plots for 19 samples, and that on the right for authentic virus neutralization activity excludes one outlier by robust regression and outlier removal (ROUT) (the same sample as in Supplementary Figure 3, resulting in plots for 18 samples. The simple linear regression analysis is conducted.

Supplementary Figure 6. Distribution of age and days elapsed from the last vaccination

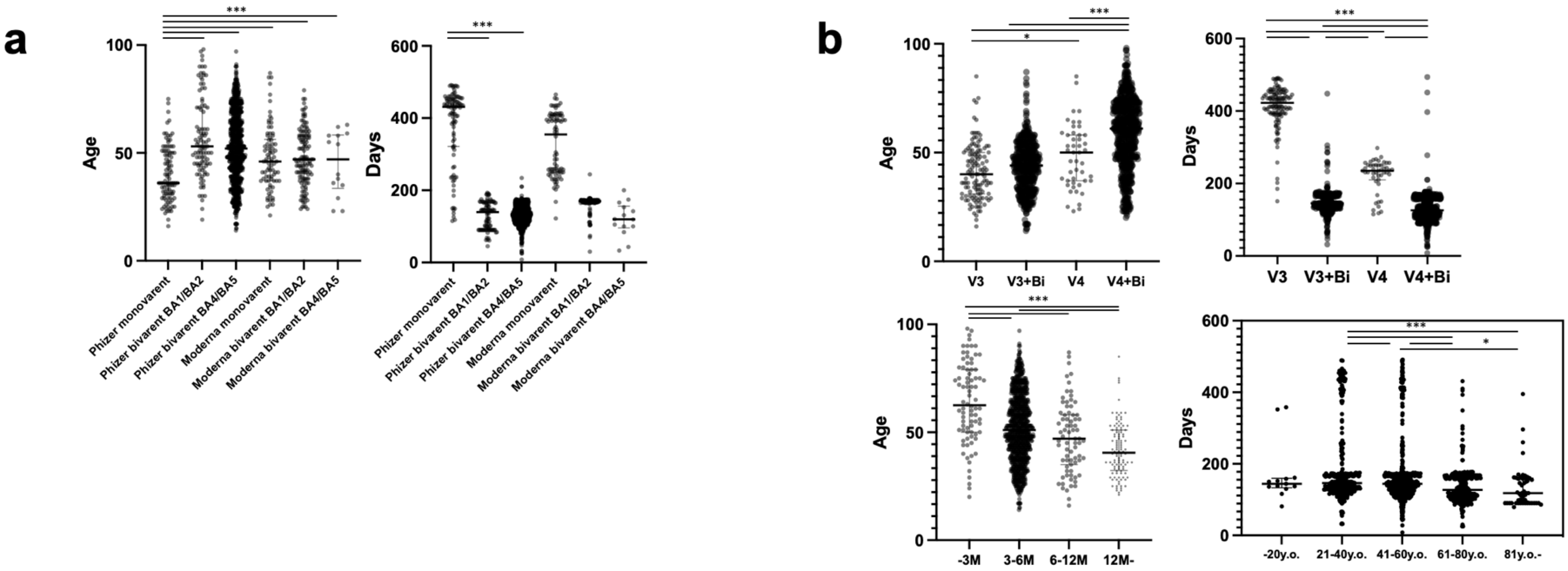

(**a**) distribution of age and time elapsed since last vaccination by vaccine type, we conducted a Tukey's Honestly Significant Difference (HSD) post-hoc test as part of an ANOVA for multiple comparisons and documented the significance of responses to the Pfizer monovalent vaccine in the figure. (**b**) distribution of age and time elapsed since last vaccination by number of vaccine doses and time elapsed, months; M, years old; y.o. We conducted a Tukey's HSD post-hoc test as part of an ANOVA for multiple comparisons*; p<0.05, **; p<0.01, ***; p<0.001

Supplementary Figure 7. Distribution of age and days elapsed from the last vaccination.

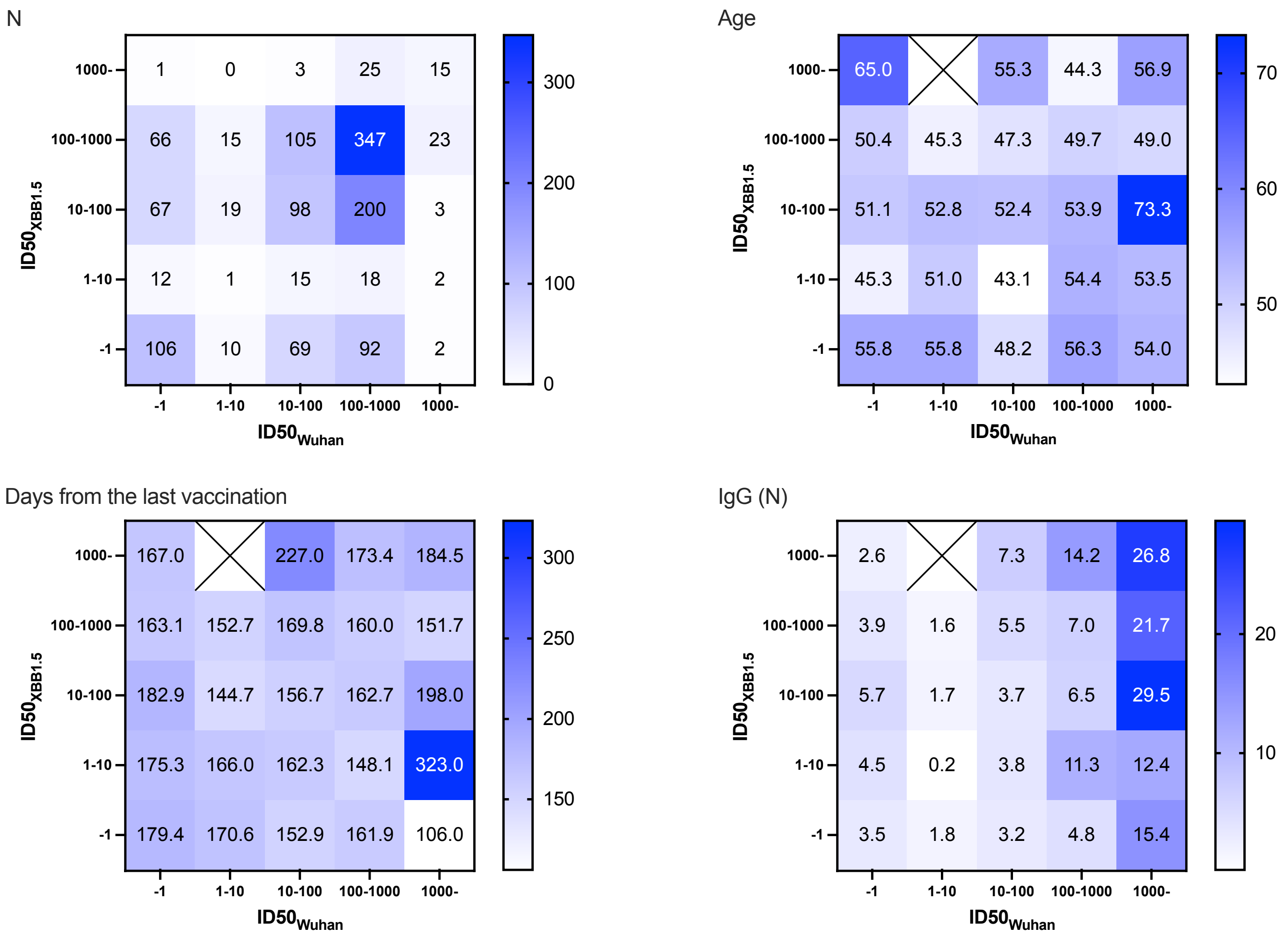

Supplementary Figure 8. Distribution of age and days elapsed from the last vaccination.

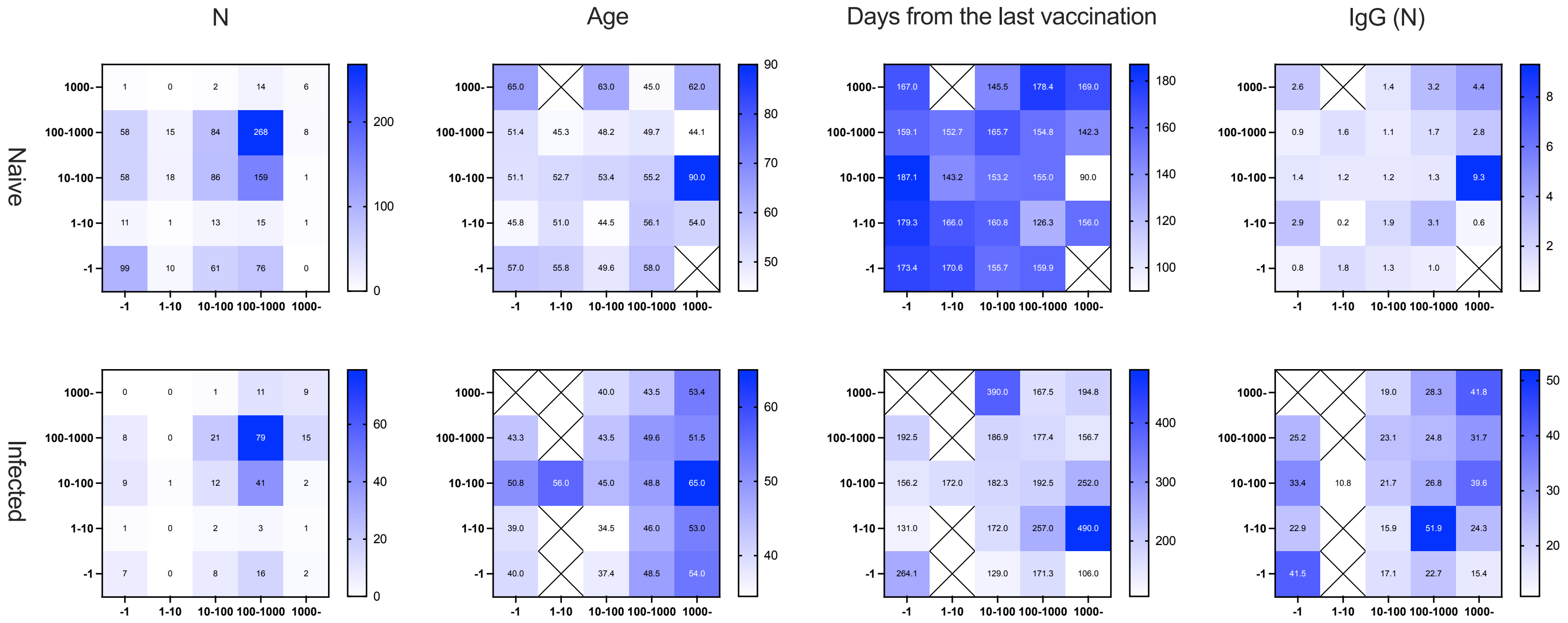

Supplementary Figure 9. Distribution of age and days elapsed from the last vaccination

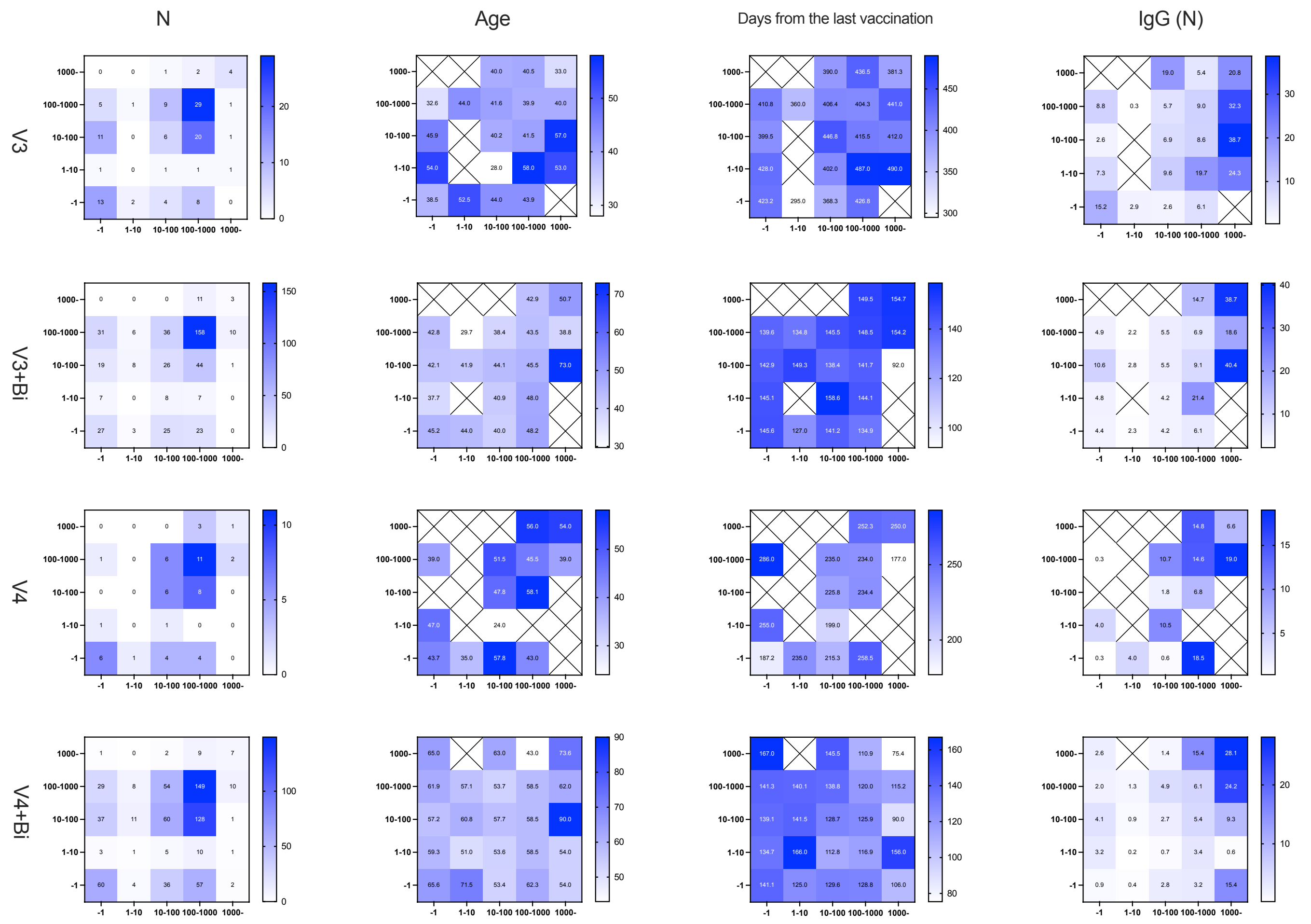

Supplementary Figure 10. Distribution of age and days elapsed from the last vaccination

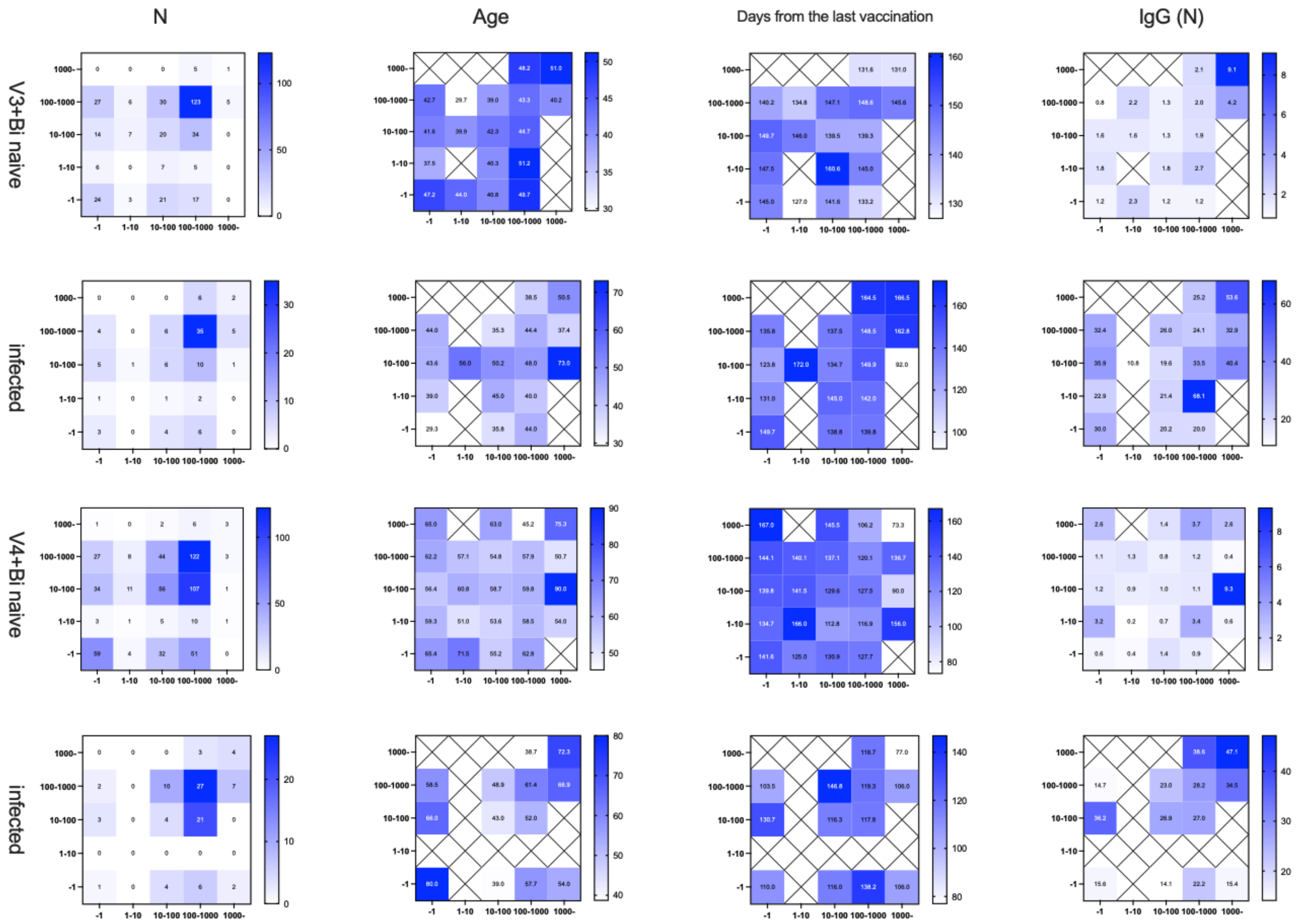

Supplement: Supplementary file 1 [file DataSheet_1.pdf]
